# Current Trends in ATRA Delivery for Cancer Therapy

**DOI:** 10.3390/pharmaceutics12080707

**Published:** 2020-07-28

**Authors:** Maria Valeria Giuli, Patrizia Nadia Hanieh, Eugenia Giuliani, Federica Rinaldi, Carlotta Marianecci, Isabella Screpanti, Saula Checquolo, Maria Carafa

**Affiliations:** 1 Department of Molecular Medicine, Sapienza University of Rome, 00185 Rome, Italy; mariavaleria.giuli@uniroma1.it (M.V.G.); eugenia.giuliani@uniroma1.it (E.G.); isabella.screpanti@uniroma1.it (I.S.); 2 Department of Drug Chemistry and Technology, Sapienza University of Rome, 00185 Rome, Italy; patrizianadia.hanieh@uniroma1.it (P.N.H.); federica.rinaldi@uniroma1.it (F.R.); carlotta.marianecci@uniroma1.it (C.M.); maria.carafa@uniroma1.it (M.C.); 3 Department of Medico-Surgical Sciences and Biotechnology, Sapienza University of Rome, 04100 Latina, Italy

**Keywords:** ATRA, delivery, targeting, DDSs, cancer

## Abstract

All-Trans Retinoic Acid (ATRA) is the most active metabolite of vitamin A. It is critically involved in the regulation of multiple processes, such as cell differentiation and apoptosis, by activating specific genomic pathways or by influencing key signaling proteins. Furthermore, mounting evidence highlights the anti-tumor activity of this compound. Notably, oral administration of ATRA is the first choice treatment in Acute Promyelocytic Leukemia (APL) in adults and NeuroBlastoma (NB) in children. Regrettably, the promising results obtained for these diseases have not been translated yet into the clinics for solid tumors. This is mainly due to ATRA-resistance developed by cancer cells and to ineffective delivery and targeting. This up-to-date review deals with recent studies on different ATRA-loaded Drug Delivery Systems (DDSs) development and application on several tumor models. Moreover, patents, pre-clinical, and clinical studies are also reviewed. To sum up, the main aim of this in-depth review is to provide a detailed overview of the several attempts which have been made in the recent years to ameliorate ATRA delivery and targeting in cancer.

## 1. Introduction

In 1881, Lunin discovered that a diet based on fat, carbohydrate and proteins was not enough to sustain the normal growth of mice, unless it was supplemented by milk.

Later on, in 1913, McCollum and Davis [1] discovered that the critical component involved in several physiological process in vertebrates was vitamin A.

It is a small molecule (286.452 Da) compared to other signaling proteins, highly oil-soluble and for this reason it is able to diffuse across the cell membrane.

Vitamin A itself is not the main bioactive mediator of its physiological function. Indeed, among its active metabolites, All-Trans-Retinoic Acid (ATRA) and 11-cis retinal play a key role in the several effects exerted by vitamin A. ATRA influences the processes of cell growth, differentiation and organogenesis [2], while 11-cis retinal have a critical role for visual function as chromophore [3].

ATRA belongs to the so-called “retinoid family”. This family has as common structural feature a β-ionone ring and a polyunsaturated side with different groups: ester (retinoic ester), carboxylic acid (retinoic acid), aldehyde (retinaldehyde) and alcoholic (retinal). The side chain is composed by four isoprenoid units with several conjugated double bonds in cis or trans configuration [4].

Several researchers have demonstrated the chemotherapeutic and chemopreventive effect of retinoid derivatives in numerous types of cancer cells [5,6,7,8]. Notably, the use of ATRA, as differentiation inducer and in combination with other chemotherapeutic agents, represents the current standard therapeutic approach used for the treatment of Acute Promyelocytic Leukemia (APL) in adults and of NeuroBlastoma (NB) in the children [9].

Despite the fact that this molecule shows a wide spectrum of functions, its use in other cancers is severely limited due to “acute retinoid resistance” [10].

Furthermore, ATRA displays poor aqueous solubility (~0.21 µM under physiological condition) [11], reduced half-life in plasma [12], thus hindering its biomedical application.

For these reasons, to overcome ATRA rapid first-pass metabolism and the issues related to its hydrophobic nature, several drug delivery systems, e.g., liposomes, nanoparticles, microspheres and microemulsions, have been investigated.

**Figure 1:**
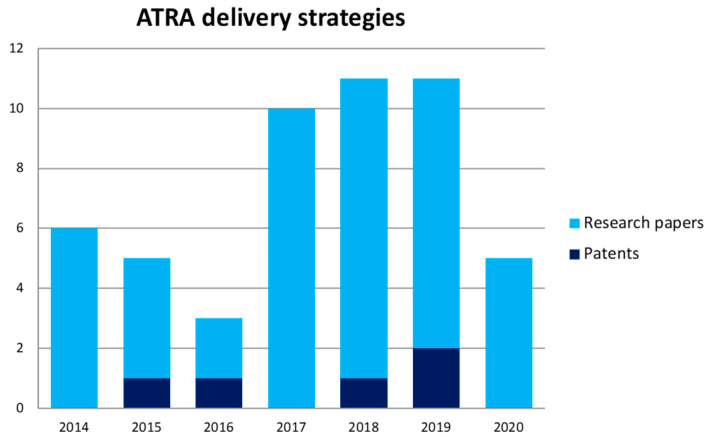
Total number of analyzed research papers and patents relative to this topic in the last 6 years until July 2020. Histograms: light blue, research paper; dark blue, patents.

In the present review we summarized the up-to-date approaches for ameliorating ATRA delivery. In order to cover the recent literature in this topic we took into account multiple databases in the research step, using the words’ combination “All-Trans Retinoic Acid”, “ATRA”, “Drug Delivery System”, “DDS” and “Cancer”. PubMed, Scopus and, Web of Science were used to find research articles whereas Espacenet was useful to review the approved patents. In the Figure 1 the total number of research articles and approved patents per year is shown, thus confirming that it is a “topic of great interest”.

All in all, in this in-depth review we will focus on some recent developments of ATRA smart delivery and targeting and its application for cancer treatment. In particular, we will evaluate the use of different Drug Delivery Systems (DDSs) to overcome the several limitations of ATRA delivery through different administration routes, facilitating drug release by passive and/or active targeting. Furthermore, we will highlight the potential advantages of ATRA-loaded DDSs within the field of cancer therapy.

## 2. From Vitamin A to ATRA

Vitamin A is a natural source of retinoids and it is assimilated from the diet to allow the normal embryonic development and body homeostasis and vision in adults. The Dietary Reference Intake Recommended Daily Amount (RDA) for vitamin A is ranging from 900–700 µg/day for adult men and women, respectively.

**Figure 2:**
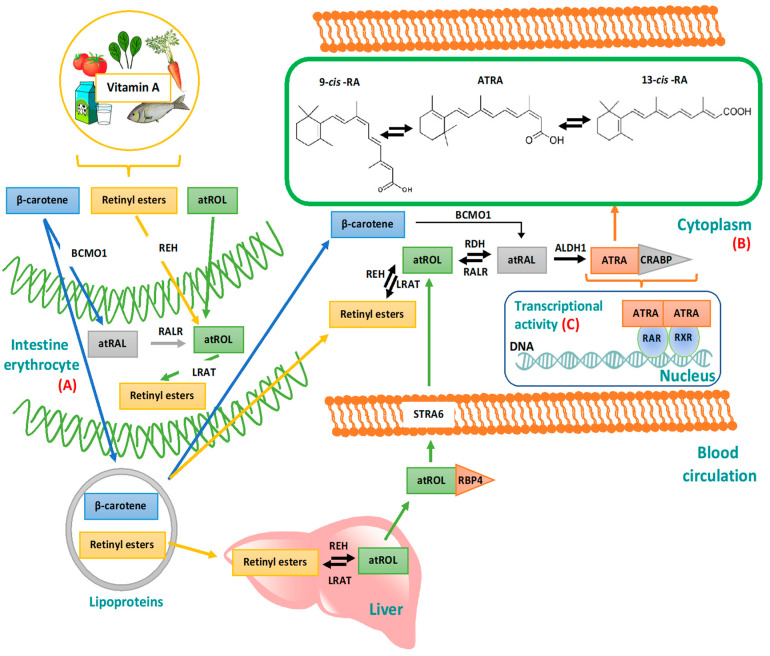
Retinoids metabolism in humans from food intake to ATRA. Graphical representation of the metabolism, transport and fate of derivatives of Vitamin A in: (**A**) gut and bloodstream, (**B**) cytoplasm, and (**C**) nucleus. Retinoids: atRAL, all-trans RetinALdehyde; atROL, all-trans RetinOL; ATRA, All-Trans Retinoic Acid. Proteins: RBP, Retinol Binding Protein; STRA6, STimulated by Retinoic Acid 6; CRABP, Cellular Retinoic Acid BP; RAR, Retinoic Acid Receptor; RXR, Retinoid X Receptor. Enzymes: BCMO1, β-carotene MonoOxigenase 1; REH, Retinyl Ester Hydrolase; RALR, RetinAL Reductase; LRAT, Lecithin Retinol AcetylTransferase; RDH, Retinol DeHydrogenase; ALDH1, ALdehyde DeHydrogenase 1.

Vitamin A metabolism in humans is shown schematically in Figure 2.

Briefly, humans can obtain vitamin A from animal-based food sources, such as milk, butter, egg and liver; whereas from plant-based diet, like spinach and carrots, it is possible to obtain high amounts of pro-Vitamin A (e.g., β-carotene) [13].

Derivatives of Vitamin A such as retinyl esters and β-carotene are transported in the bloodstream by lipoproteins. In the context of β-carotene, it is metabolized into all-trans RetinALdehyde (atRAL) by β-carotene MonoOxigenase 1 (BCMO1) directly in the cytoplasm or in the gut where it is then converted in all-trans RetinOL (atROL) by RetinAL Reductase (RALR). After absorption in the proximal small intestine thanks to bile acids, atROL is esterified in retinyl esters by Lecithin Retinol AcetylTransferase (LRAT). This reaction is important because it allows the lipoprotein-mediated transport of retinyl esters to the hepatic stellate cells in the liver, where it is stored. The ester form of Vitamin A is then hydrolyzed in retinol by Retinyl Ester Hydrolase (REH) In order to enable the systemic and intracellular transport of this compound. In this form it is able to bind the plasma Retinol Binding Proteins (RBP), thus forming the atROL/RBP complex (Figure 2A).

Cellular uptake of atROL occurs via STimulated by Retinoic Acid 6 (STRA6) receptor, an integral membrane protein which induces the dissociation of atROL from RBP, finally leading to its delivery into the cells [9].

Here, atROL is re-esterified in retinyl esters by LRAT or it binds Cellular RBPs type I (CRBPs-I). These proteins deliver atROL to metabolic enzymes responsible of converting this molecule to ATRA through two steps of reaction: (1) atROL is oxidized to atRAL by Retinol DeHydrogenases (RDHs) or cytosolic Alcohol DeHydrogenases (ADHs), which is mediated by the presence of Nicotinamide Adenine Dinucleotide Phosphate (NADP) [14]. This reaction is reversible and its direction, favorable to oxidation over reduction, depends on a family of enzymes, Short-chain Dehydrogenase/Reductase (SDR) [14], in particular, on substrate and on co-factor affinity; (2) atRAL is irreversibly oxidized to ATRA by RetinALdehyde DeHydrogenases (RALDHs or ALDHs) (Figure 2B).

In the cells, the endogenous ATRA levels are regulated by a cytochrome P450 reductase (CYP26) enzyme that degrades ATRA to 4-oxo-RA [15]. The metabolism of ATRA depends on Cellular Retinoic Acid BPs (CRABPs) which prevent its degradation. Moreover, both CRABP I and II solubilize ATRA favoring its transport in the aqueous intracellular environment up to the nucleus, where it interacts with specific nuclear receptors to start the gene transcriptional process [16] (Figure 2C).

The retinoid nuclear receptors belong to the superfamily of steroid/thyroid hormone nuclear receptors, with the function of ligand-inducible transcription factor [17]. Their structure is composed by six regions (from A to E), where the C domain is a cysteine with several DNA-binding domains and E domain contains the binding site for Retinoids. Two families of these receptors have been described: (1) the Retinoic Acid Receptors (RARs) and (2) the Retinoid X Receptors (RXRs) [18]. To date, three types of both RAR and RXR genes have been discovered in humans: -α, -β and -γ, each one for a different N-terminal protein isoform able to activate differential genes.

They have different tissue distribution and among them RAR-α is the most expressed in the body tissues. It encodes two major isoforms involved in the transcriptional regulation, RAR-α1 and -α2, that differ in their promoters (P1 and P2), mainly in the region A (A1 and A2). Both regions B and F are identical and they contain the DNA binding domain (DBD), the ligand binding domains (LBD) and the structural moieties useful for dimerization, ligand dependent trans-activation and co-repressor interaction.

ATRA induces the promoters that are responsible for the expression of different isoforms (RAR-α2, -β2 and -γ2). It is selective for RAR while 9-cis-retinoic acid (9-cis-RA, an isomer of ATRA) can bind both types of retinoid nuclear receptors [19].

Functionally, these receptors are able to act as a molecular switch depending on whether they are bound or not by their ligands. Indeed, when they are not bound, they inhibit gene expression by forming a complex with co-repressors such as Negative Co-Regulator (N-CoR), Silencing Mediator for Retinoid and Thyroid hormone receptors (SMRT) and Histone DeACetylases (HDACs). Conversely, upon ATRA binding, RAR dimerizes with RXR to form a heterodimer and the gene transcription can start. In particular, N-CoR is released and the co-activators, such as Histone AcetylTransferases (HATs) or mediators, activate the transcription of target genes starting from chromatin decompression [20].

The canonical pathway induces cell differentiation and apoptosis. Indeed, ATRA promotes cell-lineage commitment by two different mechanisms: on the one hand it up-regulates the transcription of several cell-lineage specific transcription factors which in turn activate their target genes; on the other hand it inhibits polycomb-group proteins which actively repress genes involved in cell fate decision [21]. Moreover, several studies reported that ATRA can activate both intrinsic and extrinsic apoptosis pathways [22].

Furthermore, ATRA can link other receptors such as Estrogen Receptor α (ERα), Activator Protein-1 (AP-1), Vitamin D Receptor (VDR), Liver X Receptor (LXR) and Peroxisome Proliferator-Activated Receptor (PPAR) [22]. In the last case, ATRA can activate the up-regulation of pro-survival genes, therefore an opposite function compared to the canonical pro-apoptotic one.

Moreover, ATRA can activate non-genomic pathways by regulating multiple kinase signaling pathways [23].

The selection towards one pathway or another one depends on RBPs that regulate the transport and metabolism of retinoids.

## 3. ATRA AND CANCER: Successes and Failures

**Figure 3:**
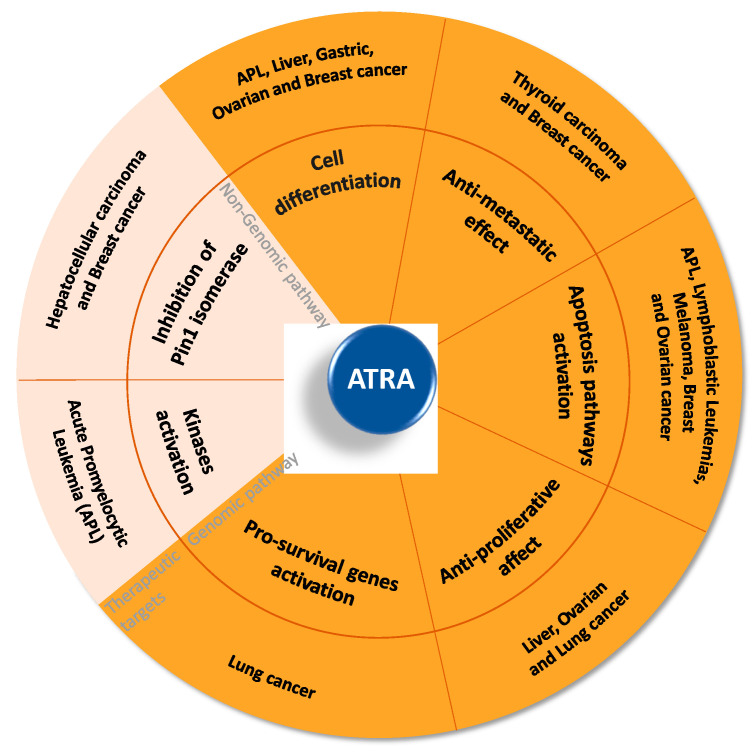
Pleiotropic effects of ATRA in cancer. The image schematically represents how ATRA affects multiple processes by genomic (orange) and non-genomic pathways (pink). Moreover, the image highlights the therapeutic targets for each process.

Given the pleiotropic effects of ATRA (Figure 3), it is not surprising that its use was proposed for cancer therapy.

The first evidence which raised the possibility of using ATRA as an anti-cancer compound came from the study conducted by Breitman and coworkers in 1980. Indeed, they documented that ATRA was able to induce in vitro differentiation of Acute Promyelocytic Leukemia (APL)-derived cells [24].

APL is a unique subtype of the genetically heterogeneous and aggressive Acute Myeloid Leukemia (AML) malignancy which is caused by the accumulation of lesions in the stem cell precursors of the myeloid lineage [25]. Specifically, APL is typically caused by the balanced translocation t (15; 17)(q24.1; q21.2) which joins the ProMyelocytic Leukemia (PML) gene (chromosome 15) with the Retinoic Acid Receptor Alpha (RARA) gene (chromosome 17) [26]. This balanced translocation creates the PML-RARA fusion gene which encodes the PML-RARalfa protein [27].

Mechanistically, ATRA is able to bind the fusion protein leading to its conformation change and its proteasome-dependent degradation, thus allowing the wild-type RAR protein to resume its normal function, finally promoting the differentiation towards the mature myeloid cell [28].

The oral administration of pharmacological ATRA for the treatment of APL-bearing patients was approved by the U.S. Food and Drug Administration (FDA) in 1995, thereby significantly improving outcomes till to gain nowadays an average overall survival (OS) near 95% [29,30]. Indeed, it represents the standard therapy still to date [31].

One positive aspect of ATRA administration is its reported low systemic toxicity despite its teratogenic effects during the first three months of pregnancy [19,32,33].

Furthermore, given that ATRA is involved in cell apoptosis and retinoic acid receptors are generally not mutated in cancer cells, expanding its use to other tumors, especially the solid ones, has captivated researchers for decades [34].

An increasing number of pre-clinical studies on several solid tumors were conducted in the past decades. Lan and coworkers documented that ATRA is able to exert anti-metastasis effects in human thyroid carcinoma cells [35], which was consistent with a previous study on human breast cancer cells [36].

More recently, it has been demonstrated that ATRA inhibited proliferation and invasion in ovarian cancer [37], liver cancer [38], and lung cancer [39].

Moreover, since ATRA plays a pivotal role in cell differentiation, it may be useful in effectively targeting tumor-initiating cells (TICs)/cancer stem cells (CSCs), which represent promising therapeutic targets to ameliorate the clinical outcome of the most lethal solid cancers [40]. Indeed, it has been demonstrated that ATRA may induce malignant reversion by promoting TICs/CSCSs differentiation towards a less neoplastically-transformed state and resulting in a more benign phenotype [41].

CSCs display self-renewal capacity and pluripotent activity [42]. These cells are involved in radiation resistance, tumor propagation to secondary organs [43] and relapse through their ability to undergo quiescence [44]. Moreover, CSCs express high levels of ATP-binding cassette (ABC) transporters which, by pumping out of cells small molecules such as cytotoxic drugs, protect themselves from drug damage and lead to multidrug resistance [45]. To date, CSCs have been associated to several solid tumors, including lung cancer [46], gastric cancer [47], liver cancer [48], ovarian cancer [49], and breast cancer [50], and it has been reported that ATRA induces differentiation of CSCs in a broad spectrum of solid tumors [51,52,53,54].

Furthermore, some studies have highlighted the role of ATRA in the up-regulation of Tight Junctions (TJs) proteins, such as occludins, claudins and Junctional Adhesion Molecules (JAMs) [55,56]. According to literature, the early stages of the invasive and metastatic cascade of tumour cells, that allows their dissemination in the human body, are caused by a loss of TJ proteins with a consequent deficit of cell-cell adhesion [57,58]. In this scenario, it is evident that ATRA exerts its anti-tumor activity also by restoring TJs. For instance, C. Moog-Lutz and co-workers [55] demonstrated that ATRA is able to dramatically increase the expression of JAML (JAM-Like) protein in NB4 APL cell, thus finally promoting growth arrest and differentiation of these cells [55]. Nonetheless, it has been recently demonstrated that sometimes the up-regulation of TJ proteins correlates with the promotion of tumor progression [59,60]. Therefore, given the dual role of TJ proteins in cancer, the function of these proteins should be taken into consideration when designing a proper therapeutic approach with ATRA.

Notably, the several performed pre-clinical studies pointed out that ATRA affected multiple cancer-driving pathways also in a transcription-independent manner [22] by the activation of several kinases, such as PKA and MAPK, which have a central role for intracellular signaling in different cell types and contexts [61,62].

Moreover, it is noteworthy that ATRA induces the degradation of the peptidilprolylil-cis/trans isomerase Pin1 by binding its active site [63]. Given that Pin1 is overexpressed in a wide range of tumors and it sustains several oncogenic pathways [64,65,66], these findings fostered the application of ATRA in the treatment of a great variety of solid tumors. Among them, ATRA-induced Pin1 ablation and the following antitumor activity have been confirmed in hepatocellular carcinoma [67,68,69] and breast cancer [70,71,72].

In spite of the promising results achieved in the pre-clinical phases for the treatment of solid tumors, ATRA-based therapies were ineffective in the clinical trials [22].

The major drawback relies on the resistance to ATRA that solid tumors could develop during carcinogenesis (intrinsic resistance) [73] or over the long-term treatment (acquired resistance) [39]. Several mechanisms are reported to be involved in the reduction of ATRA intracellular concentration in cancer cells such as increased clearance mediated by CYP26 or active efflux promoted by ABC transmembrane transporters [9]. Moreover, changes in the expression of CRPB proteins might be associated to resistance to ATRA but the published findings are contradictory given the dual role of these proteins in ATRA transport within the cells and CYP26-mediated ATRA catabolism [9]. Furthermore, altered expression or function of ATRA nuclear receptors could be involved given that their down-regulation is frequently observed [74] as well as post-translational modifications which increase their degradation [75], mutations in the ligand-binding domains [76] or de-regulation of other components of the transcription complex [77,78].

To sum up, ATRA is a promising anti-cancer compound but there are still many obstacles to its effective use in non-APL malignancies.

## 4. ATRA Delivery Strategies: What We Got in the Clinics

In addition to the above-mentioned drug resistance mechanisms, some ATRA properties may limit its clinical efficacy such as its hydrophobic nature, which does not allow parenteral administration, its susceptibility to light, heat and oxidants [11], and the short biological half-life in humans (t_1/2_ = 45 min) caused by its metabolism regulation by CYP-450 in the liver [12]. Furthermore, the current modality of ATRA administration, its variability in plasmatic concentration among patients and/or after prolonged administration [79,80] represent some of the causes which hamper an efficient ATRA delivery to tumor site.

All in all, a wide variety of studies are ongoing to ameliorate the use of ATRA, mainly focused on (i) testing it in combination with different anti-tumor compounds, in order to reduce ATRA concentration and overcome drug resistance [81], or (ii) improving its delivery for cancer treatment.

For the last purpose, the use of drug-carriers has provided an innovative approach to overcome the several limitations associated with the current drug therapy such as toxicity and non-specificity [82].

An excellent example are liposomes and, since their discovery in 1965, extensive research on them has been carried out in several areas, including in the healthcare sector where liposomes are used for several clinical products, such as Doxil^®^, DepoDur™, Ambisome^®^, etc. [83]. Liposomes are vesicular drug carriers composed by phospholipids, where both hydrophobic lipid chain and two hydrophilic head groups are structured to form a closed bilayer, surrounding an aqueous core [84]. This particular structure allows them to encapsulate both hydrophobic and hydrophilic drugs, protecting them from harsh bloodstream environment and enhance their targeting to the diseased tissues. These nano-structures are the first Drug Delivery System (DDS) successfully translated from laboratory to real-time clinical application.

In the context of ATRA delivery, Aronex Pharmaceuticals designed a product which consists in ATRA-loaded liposomes (ATRAGEN™), the unique formulation tested in clinical trials. Ozpolat and colleagues [85] analyzed the favorable liposomal ATRA (Lipo-ATRA) pharmacokinetic profile, with respect to the oral ATRA treatment, considering it effective in the treatment of APL or other responsive cancers. They enrolled 29 healthy volunteers, ranged 19–46 years. Sixteen of them were randomly assigned to the intravenously Lipo-ATRA group, while 13 were assigned to the oral ATRA group. Lipo-ATRA (90 mg/m^2^) and oral ATRA (45 mg/m^2^) were administered for 15 days. Twenty-two final subjects (11 in each group) completed the study and were evaluated. At the end of the study, the Authors calculated the area under the plasma concentration-time curve, AUC (0,∞), and the maximum plasma concentration (C_max_) of ATRA for both administration ways. They observed that oral ATRA regimen resulted in a significant decrease in the AUC (0,∞) and C_max_, which showed a reduction of 36.1% at day 9 respect on day 1 while Lipo-ATRA administration correlated with higher plasma concentrations (4.4 and 6.7-fold on days 1 and 15, respectively). In addition, liposomal formulation prevented the rapid clearance of ATRA in treated patients.

These findings suggested that intravenous administration of Lipo-ATRA was able to favor the maintenance of higher and more stable plasma concentrations of the drug, although side effects were observed as moderate and similar to the oral ATRA administration [85].

Based on these observations, in order to evaluate ATRA therapy in inducing a complete Clinical Remission (CR) in most of APL-bearing patients [86], Lipo-ATRA started to be used for APL patients treatment in some clinical trials. An interesting American trial conducted on APL patients, newly diagnosed and relapsed ones, revealed that Lipo-ATRA, as single agent, had activity in both groups [87]. Lipo-ATRA was evaluated in a total number of 69 APL patients (32 newly diagnosed, 35 relapsed and 2 oral ATRA failed) and it was administered every day until complete remission (90 mg/m^2^). In an intent-to-treat (ITT) analysis of all cases, CR rates were 62%, 70%, and 20% in newly diagnosed, first relapses-group (ATRA naive patients or cases who have not taken oral ATRA more than 1 year), or second relapses group (subsequent relapsed patients or who have taken oral ATRA during the last year), respectively. In addition, one-year survival of ITT patients was 62%, 56%, and 20% for each group respectively. However, each group of patients included a particularly high proportion of cases with a very poor prognosis. After excluding these cases, the CR rate in the 23 evaluable newly diagnosed patients increased to 87%. This ratio rose to 89% if patients showed White Blood Cell count (WBC) less than 10,000 cells/μL.

This trial was the starting point for few important considerations. Firstly, the efficiency of Lipo-ATRA depends on disease progression grade. Indeed, in newly diagnosed or in first relapse patients, Lipo-ATRA was an effective salvage agent. In contrast, no patients in second or subsequent relapse reached the CR after treatment. Secondly, the efficiency of Lipo-ATRA increases in patients with less than 10,000 cells/μL of WBC, because of their better condition in disease progression.

More recently, another clinical trial supported the ability of Lipo-ATRA in inducing a significant tumor remission in patients presenting low WBC count (less than 10 × 10^9^ /L), while it remained not effective in patients with higher WBC counts (CR rate of 92% vs 38%, respectively) [88]. Notably, Tsimberidou A.M. and her team also observed that Lipo-ATRA showed a significant efficiency as a monotherapy, thus reaching a similar CR rate observed with the use of conventional combined therapy of oral ATRA plus idarubicin [89] but avoiding all the undesirable associated side effects [88]. This last observation was encouraging towards a ‘targeted’ therapy use, in APL or in other subsets of leukemic disease.

Another clinical trial by Estey and colleagues reported the results of 12 cases scratched from a population of 18 initial subjects (ranged from 11 to 72 years) with a median WBC count at presentation lower than 10,000 cells/μL, which has been administered Lipo-ATRA as a single agent, for both remission induction and maintenance [90]. In only 3 of 12 patients was added idarubicin when PCR test remained positive or reverted to positivity, after the administration of Lipo-ATRA for 3 months from the date of initial hematologic CR. Patients received 90 mg/m^2^ of drug every day until 9 months for remission induction. Focusing on patients who had not received chemotherapy at all, 12/12 were PCR negative at 3 months from hematologic CR date, 8 were PCR-negative at 6 months, 5 at 9 months, 4 at 12 months, and 3 remained negative at 15 to 17 months. Even if the proportion of Lipo-ATRA-treated cases who will long-term require chemotherapy are, in most cases, unknown, these findings supported the important role of Lipo-ATRA administration as single agent in inducing PCR negativity in some of newly diagnosed APL patients, thus suggesting a novel first-line therapeutic approach consisting in a reduced amount of associated chemotherapeutics, contrary to what observed for oral ATRA therapy [91]. Further follow-up will be needed to ascertain the duration of tumor CRs in all patients.

More recent clinical trials proposed the use of Lipo-ATRA in addition to the conventional drug interferon (IFN) for the treatment of patients with advanced renal cell carcinoma (RCC) [92,93]. In order to evaluate the feasibility, efficacy, and biologic effects of drug combination Boorjian and colleagues treated 26 patients with Lipo-ATRA plus IFN, demonstrating that the liposomal formulation of ATRA was able to improve the effects of IFN-based therapy, contributing to a more durable response. In addition, the authors demonstrated that Lipo-ATRA also showed a good tolerance.

Lipo-ATRA was also used with success to treat patients with acquired immune deficiency syndrome (AIDS)-associated Kaposis Sarcoma. Bernstein and colleagues demonstrated that intravenous infusion of Lipo-ATRA alone, administrated at 3 doses (60, 90, 120 mg/m^2^), correlated with a significant stabilization of Sarcoma disease [94]. Further evaluations of these results are needed.

In conclusion, several clinical trials suggested how Lipo-ATRA formulation and its intravenous administration could be beneficial in cancer therapy with respect to free ATRA oral administration. Several advantages may be useful: firstly, the dosage form that can be more reliable to children who cannot easily swallow capsules and to intubated or unconscious patients, for which it would be difficult the capsules feeding. Secondly, the absorption that can be more consistent compared to patients who receive oral ATRA in combination with chemotherapy. Thirdly, the more stable blood concentration allows for more effective maintenance of therapy.

Despite these interesting results, to date ATRAGEN^TM^ did not receive FDA approval because it was not possible to identify a population of patients who could not use oral formulation.

## 5. ATRA Delivery Strategies: Moving Forward

Despite of ATRAGEN^TM^ ”failure”, the encouraging results obtained by the administration of Lipo-ATRA formulation and the strong evidence that ATRA is a promising anti-cancer compound prompted researchers to investigate novel and smart ATRA delivery and targeting strategies.

Recently, different targeted drug delivery platforms have been formulated in order to overcome the several disadvantages of ATRA treatment, such as liposomes [95], solid lipid nanoparticles [96,97], polymer nanoparticles [98] and lipid-coated inorganic nanoparticles [99]. Moreover, DDSs may be constructed in order to promote simultaneous delivery of different drugs. For instance, in several studies ATRA was delivered as an adjuvant together with chemotherapeutic [100,101] or anti-tumor drugs [102].

Interestingly, recent studies of novel ATRA delivery carriers (Table 1) are oriented towards different administration routes. Some of them have stepped into patents which are described in the last section.

### 5.1. Oral Administration

As mentioned above, ATRA represents a drug of choice for the treatment of APL. However, the effective delivery of this drug after oral administration is very challenging due to its low bioavailability, low permeability (log *P* = 6.3) and poor aqueous solubility (0.19 μg/mL) [103], thus limiting its absorption from gastrointestinal tract [104].

Microemulsions have been proposed as DDSs to improve ATRA oral absorption [104]. Microemulsions are colloidal systems composed by two immiscible liquid phases (oil and water), stabilized by surfactants; they can be divided into two groups, water in oil (W/O) or oil in water (O/W). In O/W emulsions, oil is dispersed in a continuous water phase, while in W/O emulsions, water droplets are dispersed in oil. In particular, O/W system showed the main advantage of the high-solubilizing ability of a lipophilic compound in the oil phase, dispersed in an aqueous formulation [105].

In order to improve the biocompatibility of the formulation, fish oil was used, as it represented a dietary source of long-chain omega-3 fatty acids able to reduce plasma triglycerides. Interestingly, studies testing the formulation solubility showed the capability of microemulsion to solubilize ATRA up to 10–20 mg/mL by using oleth-5 and Transcutol P in the formulation, as surfactant and co-surfactant respectively. Moreover, in vitro tests revealed a significant improvement of ATRA intestinal absorption with similar cytotoxicity between drug-loaded microemulsion and ATRA solubilized in the oil.

Another DDS employed to deliver ATRA is represented by Solid Lipid Nanoparticles (SLNs), which are composed by lipids in solid state at room temperature. SLNs combine several advantages of other DDSs such as manufacturing on large industrial scale and low cytotoxicity, similar to liposomes and emulsions, and ability to allow a sustained drug release, like polymeric nanoparticles [106].

The obtained results highlighted that a particles size, less than 400 nm, represents a key factor to reach high concentration in the gastrointestinal tract of hydrophobic drugs, by the increase of surface area and saturation solubility. These results were achieved by the use of two components in the SLNs formulation, such as Pluronic F-68 and Tween 80 [107]. Interestingly, Pluronic F-68 promoted ATRA absorption in the gastrointestinal tract due to its bioadhesion properties which allowed to increase the retention time of DDS in the targeted site. Furthermore, oral pharmacokinetic studies conducted on male rats have shown a significant bioavailability improvement of ATRA when it is incorporated into SLNs compared to free ATRA solution.

### 5.2. Intravenous Administration

Intravenous injection provides an alternative route for administration of drugs to patients unable to tolerate oral medications and offers several advantages over the oral formulation.

It has been reported that intravenous injection of ATRA-encapsulated liposomes circumvents the initial hepatic clearance following repeated oral administration [108]. Furthermore, among all administration routes, intravenous injection allows direct access to the circulatory system and instant drug action [109].

The biodegradability and biocompatibility are currently considered as key issues to determine if a DDS is suitable to be intravenously administered, to avoid the insurgence of acute adverse reactions, immunoreactivity and body toxicity. As previously mentioned, the DDSs mostly approved by FDA are liposomes and lipid-based nanoparticles [110]. In the past decades, several alternative materials have been designed and synthetized. An example is the poly (D,L-lactide-co-glycolide) (PLGA), a FDA approved polymer, biodegradable and biocompatible, widely used in biomedical field [111]. It is the compound mostly used to formulate DDSs which are under clinical trials or patented, as shown below [112]. Another example is Human Serum Albumin (HSA)-based nanoparticles which achieved great attention in pharmaceutical field due to their characteristics of non-toxicity, non-immunogenicity, biocompatibility and biodegradability [113]. In particular, albumin is a multifunctional protein carrier for drug delivery able to deliver hydrophobic drugs. Given its good tolerance in vivo, albumin was object of several clinical studies such as Albunex™ or Abraxane™.

HSA application in cancer therapy is particularly promising due to its ability to be internalized by tumor cells through gp60 pathway, thus enhancing drug distribution and bioavailability [114]. Huang and colleagues designed HSA-based nanoparticles for the co-delivery of both ATRA and Paclitaxel (PTX) in order to hinder the metastasis onset in the breast cancer [115]. These results showed that this simultaneous delivery increased the individual drug’s efficacy both in vitro and in vivo, probably due to the physical properties and pharmacokinetics changing of the two drugs in the nanoparticles. More importantly, the nanoparticles significantly inhibited the migration and invasion of cancer cells in vivo, successfully preventing cancer metastasis, reducing the activity of cancer cell Matrix MetalloProteinases (MMPs) and tumor cell Epithelial-to-Mesenchymal Transition (EMT).

Despite these interesting results, the intravenous administration opens several challenges for DDSs, as their therapeutic efficiency may be reduced by renal clearance, activation of innate or acquired immune system, specific distribution to the target tissue and cellular uptake.

**Figure 4:**
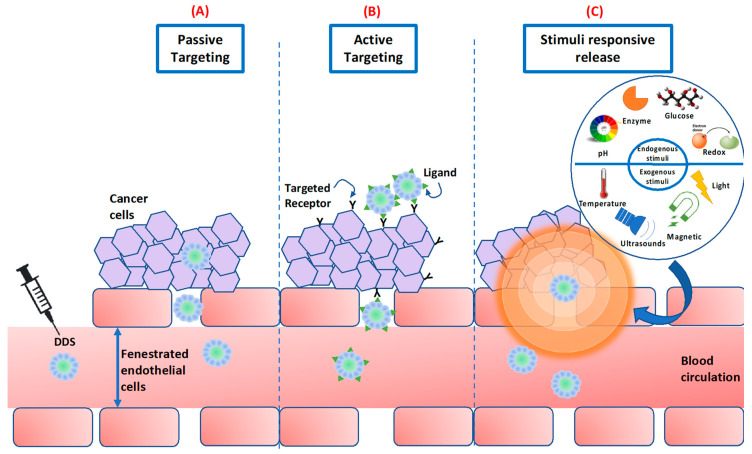
Targeting strategies of ATRA-loaded DDSs. Graphical representation according to targeting strategies: (**A**) passive targeting, (**B**) active targeting, and (**C**) responsiveness to endogenous and/or exogenous stimuli.

In the following sections these biological barriers will be described, thereby showing different types of DDS and explaining how they improve ATRA accumulation in tumors through passive or active targeting, or stimulus responsiveness strategies (Figure 4).

#### 5.2.1. Stealth Strategy

One of the most issues associated to the intravenous administration of a DDS is its rapid degradation and removal by the Mononuclear Phagocytic System (MPS) or Reticulo Endothelial System (RES), with the consequent inability of the drug carrier to reach the targeted site at the appropriate concentration and for a prolonged time [116].

To overcome this issue, the “stealth strategy” has been used by several researchers. It involves the variation of the surface properties of the carrier shell by using hydrophilic polymer, such as polyethylene glycol (PEG), polyvinyl alcohol or chitosan [117]. Currently, the use of PEGylated liposomal doxorubicin (DOXIL/Caelyx) represents an example of stealth DDS approved for clinic [118].

In this scenario, Li and coworkers developed poly(ethylene glycol)–poly(lactide-*co*-glycolide) (PEG–PLGA) polymer micelles containing Sorafenib and ATRA [119] to ameliorate the current treatment of patients bearing Differentiated Thyroid Cancers (DTCs) that de-differentiate into more aggressive malignancies [120]. The Authors investigated the antitumor effects of the micelles in vitro and in vivo. Interestingly, the proposed formulation allowed prolonged circulation time, effective delivery to the tumor site and within the tumor cells, and controlled drug release [119]. Moreover, ATRA and Sorafenib were co-loaded with miR-542-3p in PEGylated Gelucire-based SLNs for gastric cancer treatment. In vitro and in vivo results suggested the anti-tumor efficacy of the designed nanocarrier [121].

The aforementioned ATRA effect on CSCs has been well documented [122]. However, according to literature, to obtain an effective “differentiation therapy” is crucial the simultaneous elimination of both CSC and non-CSCs in the tumor, because the latter can spontaneously turn into CSCs [123,124]. For this reason, the combination of more drugs resulted in an efficacious approach for cancer treatment. In particular, several researchers demonstrated that the co-delivery of two compounds by a single DDS shows synergistic effect on cancer, while this phenomenon has not been observed with a simple physical mixture of two drugs loaded into different DDSs [125,126].

In this context, Sun and his group developed poly(ethylene glycol)-block-polylactide (PEG-b-PLA) NanoParticles (NPs) loaded with the single emulsion method of both ATRA, which induces CSCs differentiation, and DOX, which targets the differentiated ones [127]. PEG-b-PLA was used as a matrix to simultaneously immobilize both drugs with a high entrapment efficiency. Moreover, it guaranteed a slower concurrent release of molecules from NPs. In vivo studies on female NOD/SCID mice documented that NP_ATRA/DOX_ effectively increased the drugs uptake by breast CSCs and normal breast cancer cells, remarkably enhancing tumor growth inhibition and decreasing the total breast CSCs in the tumor environment, compared to free drugs, NP_DOX_ and NP_ATRA_.

However, recently studies reported some disadvantages related to the use of PEGylation, thereby leading to the so-called “PEG dilemma” [128]. These long polymer chains may prevent the binding of the targeting ligands on the DDS to the corresponding receptors, thus negatively affecting the internalization process [129]. Furthermore, PEGylation may also decrease drug release [130], and prevent endosomal escape of the DDS [131], which is necessary to avoid lysosome degradation [132]. Moreover, some clinical and animal studies reported the occurrence of immunogenic responses against PEG after systemic injection [133].

Therefore, PEGylation displays this double-edged nature which needs to be taken into account. Indeed, to overcome these issues, it has been fostered the design of nanocarriers with cleavable PEGylation [128].

A different “stealth strategy” has been proposed by Gaber et al. who employed chondroitin sulfate, an anionic hydrophilic polysaccharide, as the external shell of a hydrophobic core made of the protein Zein, containing ATRA and Etoposide. The Authors claimed that the hydrophilic shell would decrease RES recognition, thus allowing prolonged circulation time but they did not perform pharmacokinetic studies. Nevertheless, the amphiphilic copolymeric micelles were able to decrease tumor volume in vivo in larger extent than free drugs [134].

#### 5.2.2. Passive Tumor Accumulation—Enhanced Permeability Retention Effect

After entry into the systemic circulation, the DDS needs to reach the target tumor tissue.

Typically, drugs with low molecular weight can enter/exit from healthy or tumor tissues, without accumulating in them. In addition, one of the major issues to face in the solid cancer therapy is the lack of tumor selectivity of anticancer drugs. According to literature, tumor tissues are characterized by hypervascularization with a lack of lymphatic drainage and a high production of vascular permeability factors, which provokes the presence of irregular gaps between endothelial cells in the tumor vasculature [135].

In this scenario, nanotechnology can help to achieve high accumulation of drug nanocarriers in tumor tissues, avoiding the surrounding healthy tissues. Indeed, DDSs with nanometric size (between 8–100 nm) can extravasate through porous and permeable cancer vessels. The retention of nanocarriers in the tumor site is caused by reduced lymphatic drainage. This phenomenon is known as the Enhanced Permeability and Retention (EPR) effect [136] and it allows modest specificity, thus finally providing an increase of 20−30% drug accumulation in tumor site compared to normal organs [116] (Figure 4A).

To take advantage of this effect, Zhang and colleagues formulated NPs useful for the treatment of breast cancer, able to simultaneously deliver Doxorubicin (DOX), Low-Molecular-Weight Heparin (LMWH), which can inhibit the interaction of angiogenic factors such as Vascular Endothelial Growth Factor (VEGF), and ATRA [137]. Both in vitro and in vivo studies have shown the accumulation of NPs in tumor tissues via EPR effect, decreasing the typical monotherapy toxicity. Furthermore, this co-delivery system has demonstrated a higher anticancer activity in inhibiting tumor growth.

To sum up, drug delivery by using EPR effect represents a good starting point to improve drug accumulation in tumor tissues, but the particle size is not the only factor to be taken into account to determine a tumor target specificity [138].

#### 5.2.3. Active Targeting—Surface Functionalization with Specific Targeting Ligands

Drug carrier can be decorated with specific targeting ligands in order to recognize the overexpressed receptors on tumor cells [139], paving the way for novel strategies for efficient and specific targeting (Figure 4B). This strategy is often used to target TICs/CSCs. Indeed, given the role of ATRA in “the differentiation therapy”, several researchers have employed functionalized DDSs loaded with ATRA to target CSCs [140,141].

Chen and coworkers employed PLGA-Lecithin-PEG Nanoparticles (PLPNs) conjugated with both antibodies against CD44 and CD133 and loaded with ATRA (CD44/CD133-ATRA-PLPNs) to target two different populations of gastric cancer stem cells [142]. Their strategy takes advantage of the presence of markers (CD44, CD133) highly expressed on tumor cell membrane [143,144]. However, in vivo studies are necessary to evaluate the effective biodistribution of DDSs because gastric cancer stem cells represent a relatively small population compared to the total tumor mass.

Similarly, Li and coworkers employed hyaluronic acid (HA) as tumor targeting ligand [145]. This strategy was consistent with a previous study where this compound had been exploited to actively deliver ATRA and Gambocyc acid [146]. Indeed, HA is a natural polysaccharide often used due to its ability to specifically link CD44 receptors overexpressed on CSCs surface [147]. Furthermore, HA is able to form a hydrophilic layer on NP surface, protecting it from opsonization [148]. The Authors developed NPs based on cationic serum albumin by linking ethylenediamine with bovine serum albumin. These DDSs displayed good biocompatibility, very low toxicity and biodegradability due to the cationization process which maintains the protein structure and activity. Moreover, in vitro studies documented an efficient and specific cellular uptake of this formulation in B16F10 cells while in vivo studies showed that the DDS selectively accumulated in lung cancer-bearing mice and significantly inhibited tumor metastasis [145].

Among tumor antigens, beyond CD44 and CD133, CD20 is considered a good target too, especially for melanoma-initiating cells [149]. Indeed, anti-CD20 antibody-conjugated PLGA NPs were constructed for ATRA delivery and they exerted potent cytotoxic effects against CD20^+^ melanoma cells [140]. Moreover, Stauffer and coworkers formulated NPs with a fusion protein scaffold comprising apolipoprotein A1 (APOA1), for nanoparticles stability and water solubility, and a single chain variable antibody fragment (scFv) against CD20, in order to target lymphoma cells which predominantly express CD20 [150]. Moreover, CD19 or CD30 may be exploited to target B-cell malignancies inefficiently targeted by anti-CD20 antibody [151].

#### 5.2.4. Cellular Uptake and Endosomal Escape

Once reached the target tumor tissue, DDSs should be internalized by tumor cells.

Several factors can contribute to enhance the interaction between a DDS and the cell membrane. Narvekar and coworkers constructed Polymer-Oil Nanostructured Carriers (PONCs) where ATRA is dispersed in the oil phase within the polymeric matrix. These studies showed that ATRA-oil droplets efficiently permeated the lipid bilayer of the plasma membrane compared to the free drug, probably due to the ability of oil in promoting drug uptake into the cancer cells, thus increasing its permeation across the lipid membrane [152].

Moreover, beyond passive transport of a lipophilic drug, the DDS surface can be functionalized to promote endocytosis-mediated uptake and the following endosomal escape [153]. Several researchers have proposed different approaches to promote cellular uptake of a DDS, as discussed in the below sub-sections.

##### Positive Charge Surface

In order to promote the interaction with the negatively charged compound of cellular membrane, several researchers developed liposomes using cationic lipid, e.g., 1,2-Dioleoyl-3-trimethylammonium-propane (DOTAP). This compound is a synthetic cationic lipid, bearing a positively charged head group, which shows high affinity with membranes. This method allows to enhance the internalization and delivery of DDS into cells [154]. Previous in vitro and in vivo studies by using different cationic liposomes highlighted their potential in the treatment of cancer.

ATRA-loaded cationic liposomes have been demonstrated to enhance anti-lung cancer activity on A549 human lung cancer cell lines and displayed also an anti-lung metastatic activity on in vivo metastatic mice model [155,156]. More recently, Grace and coworkers formulated cationic liposomes to deliver ATRA to lung cancer-bearing mice [157]. The Authors compared the effect on cancer-dependent variation of mouse weight following tail vein injection of ATRA loaded-liposome or free ATRA solubilized in olive oil. It is well known that cancer-bearing mice are characterized by anorexia, malabsorption and cachexia with a notable loss of body weight [158]. The obtained results showed a persistent and enhanced therapeutic effect on mice affected by lung cancer after 30 days, with a low decrease of body weight, thereby confirming an increase of drug uptake for mice treated with ATRA-loaded cationic liposomes compared to free ATRA. Furthermore, pharmacokinetic studies have demonstrated a higher half-life (t_½_ 14.8200h), maximum concentration value (C_max_ 0.66 mg/mL), and a lower clearance rate (CL 46.6061 mg/mL/h) for ATRA-loaded liposomes compared to the mice treated with free ATRA (t_½_ 13.2205h, C_max_ 0.29 mg/mL, and CL 136.2725 mg/mL/h). In addition, the presence of carboxylic acid on ATRA molecule structure seems to facilitates its packing efficiency into the DOTAP liposomes. The Authors adjusted the cholesterol concentration (to modulate the bilayer rigidity) into the liposomal formulation in order to obtain a high percentage of the encapsulated drug. The entrapment efficiency of ATRA observed in these studies was around 92%, significantly higher than other reported nano-formulations loaded with the same drug [159].

Unfortunately, if the increased efficacy of a DDS with positively charged surface has been well documented, these studies need further elucidation as they have shown a strong immune response [145].

##### Proton-Sponge Effect

Several studies reported that DDSs are internalized by endocytosis, thus following the endosomal route till lysosomal compartment [160], where they are degraded [161]. Several strategies have been developed to induce endosomal escape, among them the so-called “proton-sponge effect” which relies on the capacity of the DDS to bind protons (H^+^), thus inhibiting the drop in pH. As a result, even more protons are pumped into the endosome accompanied by chloride counter ions and water molecules which increase osmotic pressure and lead to endosomal lysis [162].

In this context, Mu and coworkers formulated dendrisomes co-loaded with both ATRA and docetaxel for differentiation therapy of breast cancer. In particular, dendrisomes have been functionalized with distearoylphosphatidyl ethanolamine polyethylene glycol (DSPE-PEG2000) to avoid RES. In addition, the latter is conjugated with a peptide, D-type hexa-arginine, to enhance the cellular uptake by breast CSCs. The designed dendrisomes were able to bind protons (H^+^) and displayed buffering capacity in acidic environment such as the lysosomal compartment, thus enhancing the endosomal escape and lower accumulation in the lysosomes [163].

#### 5.2.5. Stimuli-Responsiveness

Stimuli-responsive DDSs have been developed in order to ameliorate the targeting and to achieve rapid drug release. They are “smart carriers” specifically formulated with compounds able to change their chemical or physical characteristics in response to internal stimuli of tumor environment such as temperature, pH, proteases or redox potential, or external stimuli, like light, heat, electric/magnetic field or ultrasound [164] (Figure 4C).

Typically, cancer cells are able to generate energy by glycolysis in hypoxic environment, then the extracellular environment of tumor is characterized by a lower pH (6.5–6.8) compared to normal tissues (pH 7.4) [165,166]. In order to take advantage from this effect, pH-responsive DDSs have been exploited in anticancer research and specifically to facilitate ATRA release in pH-controlled manner [167].

In this scenario, Zhang and coworkers formulated “dual responsive” drug delivery system, pH- and redox-responsive nanoparticles to co-deliver ATRA and Paclitaxel to the specific tumor site. The Authors developed a pH-responsive DDS in order to allow a surface charge switch from negative (−16 mV, pH 7.4) to positive under tumor slightly acidic microenvironment (+16 mV, pH 6.5), thus reducing the nonspecific protein adsorption under blood circulation and promoting cellular uptake once the DDS has reached the target tumor site. Moreover, they introduced disulfide bonds to accelerate drug release in a high reducing environment [168] such as cancer cell cytoplasm due to the intracellular high concentration of glutathione [169,170]. Interestingly, in vivo studies on human lung cancer A549 tumor-bearing nude mice showed that the simultaneous rapid release of the two drugs significantly enhanced the antitumor efficiency [168]. Furthermore, similar strategy of introducing a sensitive bond was exploited also in another study where it induced a burst release of ATRA and Paclitaxel in vitro [171].

More recently, it has been reported the anti-angiogenic effects of ATRA grafted Poly Beta-Amino Ester (PBAE) nanoparticles on 3D collagen-cytodex model. The Authors claimed that ATRA-loaded nanoparticles can release drug in pH-sensitive manner, thus supporting the notion that ATRA could be faster released at tumoral pH [172].

Beyond accelerating drug release, pH-responsive DDSs may be useful also to overcome the above-mentioned issues associated with the so-called “PEG-dilemma”.

Given that it has been fostered the design of nanocarriers with cleavable PEGylation [128], Han and coworkers constructed pH-responsive PEG-detachable polyethylenimine (PEI)-coated gold nanoparticles (AuNPs) which “experienced” PEG detachment followed by DDS internalization only in the more acidic tumor microenvironment. They designed this DDS for the co-delivery of ATRA and a small interfering RNA against HSP47 to induce Pancreatic Ductal AdenoCarcinoma (PDAC) microenvironment remodeling [173]. Upon abnormal activation, Pancreatic Stellate Cells (PSCs) produce a thick extracellular matrix which hinders an effective drug delivery, thus limiting the anti-tumor efficacy of chemotherapeutics such as Gemcitabine [174]. Notably, they investigated whether their designed DDS may induce stromal modulation in vitro, in a three-dimensional (3D) PDAC stroma-rich tumor spheroid model, and in vivo studies, thus obtaining encouraging results which were consistent with effective Gemcitabine-free treatment in two murine models [173].

As regards the use of stimuli-responsive DDSs triggered by external stimuli, they offer the possibility to obtain a specific drug release or activation at a specific time and location.

It has been demonstrated that magnetic nanoparticle-based drug delivery systems are an auspicious alternative to fight the limitations of classical chemotherapies. In general, magnetic nanoparticles can be targeted to tumor site by exploiting magnetic field and they have a reactive surface which can be modified with biocompatible coatings, such us dendrimers, branched molecules which adopt a spherical morphology [175]. Yalçin and colleagues [176] described the advantages of polyamidoamine (PAMAM) Dendrimer-coated Magnetic iron NanoParticles (DcMNPs) to transport Gemcitabine and ATRA combination therapy, in order to overcome the aforementioned chemotherapy resistance of PDAC caused by the fibrotic products of PSCs. The Authors demonstrated that nanoparticles were successfully taken up by pancreatic cancer and PSC cells, where both drugs were released in lower pH condition.

Moreover, this kind of DDS allows to overcome the issue of premature drug release before reaching their targeted site [177].

For the treatment of glioblastoma (GBM), Lu and colleagues constructed DSPE-PEG_2000_ nanoparticles decorated with CARD-B6 peptide and loaded them with three different drugs, ATRA, DOX and Combretastatin A4 (CA4) [178]. B6 is a peptide with high affinity for transferrin receptors, thus allowing the entry of the DDS into the brain through the blood-brain barrier. Poly β-amino ester (PAE) was used as pH-sensitive tool to allow drugs release in GBM environment and both endosomal and lysosomal escape. In addition, azobenzene (AZO) bonds were employed given their ability to break themselves under hypoxic condition, allowing a controlled release. To trace the accumulation of the DDS into the GBM, nanoparticles were also loaded with SuperParamagnetic Iron Oxide Nanocubes (SPIONs) which allow the traceability of drug carrier by Magnetic Resonance Imaging (MRI). The designed DDS showed a long circulation time compared to free drugs; B6 peptides effectively improved their ability to reach the tumor site traced by MRI technique. After DDS tumor accumulation, PAE breaking provoked the release of CA4, then DOX and ATRA were simultaneously released due to the AZO breaking.

Another attractive DDS able to recognize external stimuli and to enhance the drug ATRA delivery was described [179]. Li and coworkers formulated nanodiamonds loaded with both ATRA and DOX for the treatment of both liver and breast cancers. Under ultrasound effect, these DDSs were able to trigger the reversible opening of tumor endothelial tight junctions by sonoporation and cavitation mechanisms [180,181]. Furthermore, in order to increase the drug retention in the tumor site, nanodiamonds were employed as nanocarriers due to their capability to bypass the drug efflux mechanisms [182]. Both in vitro and in vivo results documented that the proposed DDS could effectively enhance the intracellular retention of both delivered drugs in the tumor site, after ultrasound application. Furthermore, a significant inhibition of tumor growth was assessed with the consequent increase of survival of mice affected by tumors.

Recently, combining internal and external stimuli in a single system was described to obtain a synergic therapeutic effect in the targeted site [183].

In this scenario, Jia and colleagues used this approach to improve the bioactivity of ATRA and the site-specific antitumor therapy in breast cancer contest [184]. This DDS exploited the photothermal and photodynamic ability of Indocyanine green (ICG), a near infrared dye, to generate reactive oxygen species (ROS) and heat after Near InfraRed (NIR) laser irradiation. They generated nanoparticles encapsulating ICG dye with coumarin-containing ATRA (AC), via linking ATRA with 7-hydroxy-4-trifluoromethyl coumarin (HTCM) by ester bond for the first time. Coumarin, namely benzopyrone, was chosen for its anticancer activity. AC and ICG-containing nanocarrier was modified with the targeted ligand cyclic (Arg-Gly-Asp-D-Phe-Lys) (cRGD) peptide, in order to increase the accumulation of drugs through the recognition of the overexpressed integrin αvβ3 on tumor cell surface. After internalization, the AC/ICG-TNPs nanoparticles rapidly release drug in the mild acidic microenvironment of lysosome. Using this approach, the photothermal/photodynamic therapy upon NIR irradiation was added to Coumarin and ATRA chemotherapeutic treatment. Apoptotic studies on MCF-7 and MDA-MB-231 human breast cancer cells demonstrated an effective antitumor activity of the designed carriers.

The approach of combining photodynamic therapy (PDT), photothermal therapy (PTT), and chemotherapy was also used by a Chinese group who realized nanocarriers covalently conjugating ATRA with a small dye molecule diketopyrrolopyrrole (DPP-ATRA), which is functional as effective photothermal agent, photosensitizer agent and drug carrier [185]. The soluble nanoparticles selectively accumulate in tumors, release chemotherapy drug under lysosome acidic conditions, and exhibit heat and ROS generation after low power xenon lamp irradiation.

### 5.3. Inhalable Administration

Despite the numerous favorable effects of ATRA in the treatment of lung cancer, this compound has shown to be ineffective in clinical trials done so far, due to the poor drug persistence/stability in blood circulation and the issues related to reach the target site.

In order to enhance pulmonary drug deposition and provide a better patient compliance with less systemic toxicity, pulmonary administration of DDS represents a promising route to deliver drugs directly to the lungs [186]. Given its lipophilicity, free ATRA cannot be aerosolized. For this reason, the formulation of a drug carrier as a dry powder inhaler has been suggested as a possible strategy to reach lung tissue and facilitate its uptake by cancer cells.

In this scenario, the potential of liposomes vehiculating ATRA for the treatment of lung cancer has been well documented in literature. Liposomes are able to incorporate lipophilic drugs like ATRA, allowing the direct targeting to the aerodigestive tract by aerosol administration. The main advantage of the administration through aerosol is the uniform drug deposition in the respiratory tract, thus determining higher local dose compared to the systemic administration.

Mehta and coworkers compared the toxic effect of both free ATRA and ATRA loaded-liposomes in CD-1 mice. These studies highlighted that mice can tolerate a dose of free drug up to 25–30 mg/kg of body weight. On the contrary, if ATRA is encapsulated in liposome, this dose can be incremented up to 120 mg/kg, probably due to a better drug distribution in target tissues [187].

The application of liposomes as drug delivery system by aerosol route was encouraging, however their use exhibited several issues including storage instability due to oxidation processes, the leakage of loaded drug, the expensiveness of synthetic phospholipids and the variable purity of natural phospholipids [188]. To overcome these problems, researchers have developed an alternative liposomes-like structures that are composed by non-ionic surfactants, called niosomes [189]. These vesicles are able to entrap hydrophilic/hydrophobic drug and prolong drug circulation as liposomes. Moreover, they show high chemical stability, reproducible production and low cost of manufacturing.

Therefore, Desai and colleagues prepared various ATRA-loaded niosomes composed by Tween 80 and different Spans (20, 40 and 60), in order to select an optimal formulation in terms of drug entrapment efficiency, leakage after nebulization and nebulization performance [190]. In particular, in vitro studies were carried out by using a nebulizer: from the aerosol produced from the niosomal formulation upon nebulization, it has been monitored the size distribution and ATRA entrapment efficiency on each stage of an Anderson cascade impactor. The obtained results documented a good drug entrapment into the aerosolized droplets with appropriate size useful for inhalation therapy, making them a promising ATRA delivery system for lung cancer treatment.

Another interesting DDS to deliver ATRA into lungs by inhalable administration are Hybrid core-shell Lipid-Protein NanoParticles (HLPNPs), formulated in order to overcome the several limitations of lipid nanoparticles, such as physical stability and the possibility to lose the entrapped drug, but maintaining their advantages of biocompatibility and biodegradability [191]. The external shell of these structures is composed by the natural hydrophobic protein Zein which provide another compartment to load an additional drug and to have a functionalizable surface aimed at increasing nanoparticle stability and sustaining drug release characteristics. Specifically, HLPNPs were loaded with two different lipophilic drugs, ATRA and Genestein, a potent tyrosine kinase inhibitor. The Authors coupled biotin to nanoparticle surface to increase internalization into cancer cells for an effective delivery which was consistent by in vitro obtained results. Furthermore, to provide a deeply drug deposition into lungs, they produced inhalable dry powder nanocomposites to test the therapeutic effects of HLPNPs in lung cancer-bearing mice, thereby documenting a promising treatment option [191].

All in all, by comparing the effects of free drugs and DDS through both intravenous and inhalable administrations, it has been confirmed that pulmonary administration could be preferred rather than systemic delivery [186].

### 5.4. Alternative Administration

A localized drug delivery provides the use of a lower dosage of the drug and reduced drug concentration in the serum, thereby allowing the reduction of systemic side-effects.

To this purpose, the ability of ATRA to inhibit the proliferation and to induce apoptosis of glioblastoma cells is well-documented [192,193]. However, ATRA therapeutic effects after systemic administration, were inefficient essentially due to its low transport through the blood-brain barrier and its rapid in vivo metabolism by CP450 enzymes [194]. As a result, Mirani and colleagues developed ATRA-loaded polymeric microspheres, a DDS capable of prolonged release, based on the drug-eluting hydrogel mesh manufacturing [195]. In vitro studies indicated a controlled release of ATRA up to three weeks based on the mesh porosity and polymer concentration. The use of hydrogel constructs showed the advantage of increasing drug uptake by immobilizing the DDS close to tumor site. This approach supposed a brain implantation of the 3D bioprinted hydrogel preventing its dislocation by the cerebrospinal fluid.

To overcome the poor entry of ATRA through the blood-brain barrier [196], Jones and coworkers [197] suggested the local use of porous poly (1,8-octanediol-*co*-citrate; POC) wafer. They demonstrated that this DDS was able to solve the issue of the unstable nature of ATRA by decreasing both the processes of isomerization and degradation. Furthermore, given that POC wafers slowly released ATRA, they had a longer lasting effect on U87MG cell line with respect to single-dose free ATRA, thereby providing a long-term treatment option for glioblastoma multiforme (GBM).

Despite POC wafers represent promising approaches to allow a controlled and localized brain delivery of ATRA, further in vivo studies will be required to evaluate their efficacy and biocompatibility the complex physiological environment of the brain [198].

**Table 1:** ATRA delivery systems in cancer. Critical summary of analyzed studies on different DDSs grouped by tumor type.

Tumor	ATRA Delivery System	Model	Pros	Cons	Ref
**Acute Promyelocytic Leukemia**	ATRA-loaded microemulsion O/W	Porcine intestinal membrane	Oral delivery of ATRA to enhance drug bioavailability and intestinal absorption	Only in vitro studies	[104]
	DOX-loaded LMWH–ATRA nanoparticles (DHR nanoparticles) negatively charged	Cell lines: HL-60 and MCF-7; mouse model	Lower risk of bleeding and thrombocytopenia/selective uptaking endocytosis mediated		[137]
	ATRA-loaded in Cholesteryl Butyrate Solid Lipid Nanoparticles	Cell lines: HL-60, Jurkat, and THP1	High encapsulation efficiency over and enhanced anticancer activity when compared to the free ATRA	Only in vitro studies	[96]
**Breast cancer**	ATRA-loaded Pluronic F127 micelles	Cell lines: 4T1, MDA-MB-231, EMT6, and BT474; Mouse model	Biocompatibility, high ATRA loading content and synergistic effects with Cisplatin	No biodistribution studies	[98]
	Human serum albumin (HSA)-based nanoparticles for the co-delivery of ATRA and Paclitaxel (PTX)	Cell line: 4T1	Increase of individual drug’s efficacy both in vitro and in vivo, inhibition of the migration and invasion of cancer cells in vivo (reduction of cancer cell MMPs activity and of EMT process)		[115]
	Hyaluronic acid (HA) nanoparticle with an inner hydrophobic core containing ATRA and the anticancer drug Gambogic acid (GA)	Cell lines: MCF-7 and KB31	HA receptor-mediated endocytosis improves the internalization into the tumor cells		[146]
	Nanoparticles co-delivery strategy of an ATRA and DOX based-therapy	Cell line: MDA-MB-231	Selective uptaking		[127]
	Nanoparticles encapsulating ICG dye with coumarin-containing ATRA (AC), modified with the targeted ligand cyclic (Arg-Gly-Asp-D-Phe-Lys) (cRGD) peptide on the surface	Cell lines: MCF-7 and MDA-MB-231	Combination of photodynamic therapy (PDT), photothermal therapy (PTT), and chemotherapy	Only in vitro studies	[184]
	Amphiphilic zein-chondroitin sulfate (ChS)-based copolymeric micelles containing ATRA/Etoposide	Cell line: MCF-7; mouse model	Enhancing internalization in vitro and reducing tumor volume, decreasing proliferation, and promoting necrosis in vivo	No PK and biodistribution studies	[134]
**Gastric cancer**	CD44/CD133 antibodyconjugated ATRA-loaded nanoparticles	Cell lines: MKN-45 and NCI-N87	Specific target of cancer stem cells by using membrane markers	Difficulty reaching the targeted site in vivo	[142]
	ATRA/Sorafenib/miR-542-3p co-delivery in PEGylated Gelucire-based Solid Lipid Nanoparticles	Cell line: MGC-803; mouse model	Enhanced anti-tumor efficacy of drug co-loading	No biodistribution studies	[121]
**Glioblastoma**	ATRA-loaded poly(diol citrate) wafers	Cell line: U87MG	Long-term treatment in vitro and reduced ATRA isomerization and degradation	Duration of release in vivo is not known	[197]
	3D bioprinted hydrogel mesh loaded with ATRA	Cell line: U87MG	Controlled release and immobilization of DDS close to tumor site	Biocompatibility of the construct in the brain in vivo	[195]
	CARD-B6 NPs loaded with ATRA, DOX and CA4	Cell line: U87MG; Mouse model	Controlled release by using different peptide tools and a tractable DDS by MRI		[178]
**Liver cancer**	Poly(amidoamine) (PAMAM) dendrimers	Cell line: HepG2	pH-responsive DDS and enhanced cellular uptake	Only in vitro studies	[167]
**Lung cancer**	ATRA/Genestein-loaded hybrid lipid nanocore-protein shell	Cell line: A549; Mouse model	Stable inhalable dry powder		[191]
	ATRA/Paclitaxel-PEG-b-PBLA micelles (pH and redox dual-responsive)	Cell line: A549; Mouse model	Prolonged circulation time, reduced nonspecific protein adsorption effective delivery to the tumor site and within the tumor cells, controlled drug release, and negligible systemic toxicity	No biodistribution studies	[168]
	DOTAP liposomes loaded with ATRA	Mouse model	Higher half-life, Cmax and a lower CL of ATRA loaded liposomes compared to the mice treated with free ATRA.	Strong immune response	[157]
	ATRA-loaded niosomes		Inhalable DDS to enhance drug localization in the targeted site	Only in vitro studies	[190]
**Lymphoma**	ATRA nanoparticles constituted by a fusion protein scaffold comprising apolipoprotein A1 (APOA1) and a single chain variable antibody fragment (scFv) against CD20	Lymphoma	Targeted therapy thanks to selective uptake	Only in vitro studies	[150]
**Melanoma**	Polymeric micelles of hyaluronic acid – ATRA for the co-delivery of Paclitaxel and ATRA	Cell line: B16F10; Rat model	Redox-responsive drug release and higher CD44-dependent cellular uptake in vitro, and prolonged circulation time	Antitumor efficacy of the constuct is not known in vivo	[171]
	CD20-antibody conjugated PLGA nanoparticles	Cell lines: A375 and WM266-4	Better targeting and stronger inhibitory effects against melanoma-initiating cells (CD20+) with respect to CD20- cells	Only in vitro studies	[140]
	Lipid-coated Hollow Mesoporous Silica Nanoparticles-ATRA/Doxorubicin/IL-2	Mouse model	Excellent encapsulation capacity, satisfactory stability, favorable biodistribution and low systemic toxicity		[99]
**Ovarian cancer**	Polymer-oil nanostructued carrier (PONC)	Cell line: SKOV-3	Controlled and sustained release profile, biological stability and increased cellular uptake by efficient drug permeation	Only in vitro studies	[152]
**Pancreatic ductal adenocarcinoma**	PEGylated polyethylenimine-coated gold nanoparticles for the co-delivery of ATRA and siRNAHSP47	Cell lines: Pancreatic cancer primary cells; Mouse model	pH-responsive DDS, stability in the systemic circulation, negligible system toxicity, and effective accumulation in the tumor site	Quick clearance of the DDS	[173]
	Polyamidoamine (PAMAM) dendrimer-coated magnetic iron nanoparticles (DcMNPs)	Cell lines: ductal pancreatic cells and pancreatic stellate cells (PSCs)	Magnetic nanoparticles can be targeted to tumor site in a magnetic field and they successfully taken up by pancreatic cancer and PSC cells	Only in vitro studies	[176]
**Thyroid cancer**	ATRA/Sorafenib-loaded (PEG–PLGA) polymeric micelles	Cell line: FTC-133; Mouse model	Prolonged circulation time, effective delivery to the tumor site and within the tumor cells, controlled drug release, and negligible system toxicity		[119]

### 5.5. Patents

Given the large number of ATRA delivery approaches which have been discovered in the past few years, some of them have stepped into patents but not tested in the clinic trials yet. Here, we included some interesting inventions suitable to be further evaluated for new delivery ATRA approaches (all collected in Table 2).

A group of three American inventors developed a new ATRA derivate patent suitable for topical delivery (Patent n° WO2016210087A1; US2018185513A1). It included a polymer conjugated with all-trans retinoic acid prodrug (PATRA), covalently bound to the polymer by a hydrolysable linker or a salt, thus allowing ATRA solubility in water. When hydrated, PATRA formed a nano-fibers that agglomerated in submicron scale nanoparticles, as a result of the hydrolysis of the hydrolysable linker. In vitro, it has been demonstrated that ATRA release from the polymer was sustained for up to ten days in the site of administration. The skin delivery was evaluated in vivo by using an explant pig dermis showing a four-fold increase in drug accumulation within the dermis and a minimal inflammatory response compared to conventional ATRA therapy. The carriers used included mineral oil, propylene glycol, polyoxyethylene compound, emulsifying wax and water. In certain embodiments, PATRA could be administered orally, presented in capsules, sachets or tablets each containing an amount of active ingredient. In another embodiments, this invention could be suitable for aerosol, rectal, pulmonary and parenteral administration in addition to the topical one. This formulation could represent a substantial method to efficiently control the delivery of ATRA.

Gilbert and colleagues optimized an aerosol-delivered liposomal-ATRA as an efficient and nontoxic way of delivering higher levels of drug for the upper aerodigestive tract and lung cancer treatment (Patent n° US6334999B1). As described in the previous section, since free ATRA cannot be aerosolized, due to its lipophilic properties, the retinoid was incorporated into liposomes, which were resuspended as an aqueous suspension to be aerosolized and inhaled. In one embodiment of the invention, the liposomes were composed of dipalmitoylphosphatidylcholine (DPPC) and stearylamine (SA) in a typical formulation of 9:1, and the retinoid was incorporated into liposomes at a drug:lipid ratio of 1:10. The liposomes diameters in the aerosol felled within the 100 to 1000 nm range. Nebulization was necessary to obtain aerosol particles with mass median aerodynamic diameters of 1 to 3 microns. In vivo, ATRA retained its biological activity as revealed by the increased expression and activity of alveolar macrophages enzyme tissue-type transglutaminase (TGase), which was induced by retinoic acid treatment. Regardless, further evaluation on animal models should provide support for using this mode of delivery in clinical settings. This invention could give the possibility to ATRA to be deposited more uniformly over the respiratory tract and to be locally administered, thus exceeding the levels achieved by systemic administration with minimal toxicity.

Giannoukasis and his group, designed a particle formulation of ATRA for the treatment of autoimmune diseases, such us type I diabetes mellitus (Patent n° WO2015109245A1; US2016338984A1; US10105334B2). The particular formulation included a particle comprising ATRA encapsulated within a polymeric matrix, and the transforming growth factor beta (TGFβ) absorbed on a surface of the particle. The encapsulation was accomplished by an emulsification process using PLGA as a drug dispersal matrix. The ability of particles to reverse diabetes symptoms was tested in mice model: animals receiving PLGA-Ni/ATRA/TGFβ particles showed significantly lower glucose levels compared to the control mice.

A group of three Chinese inventors developed a patent which provided ATRA quasicrystal and liposome preparation (Patent n°CN109364027A). The preparation had a high stability, a uniform drug release and an encapsulation efficiency of 94–100%. The liposomes included lecithin, cholesterol, and pegylated phospholipids, while the internal aqueous phase comprised an aqueous solution of calcium acetate. The effects of this trans-retinoic acid liposome were observed on colon cancer cells through the evaluation of (i) the ability of the myeloid-derived suppressor cells (MDSCs), to differentiate into mature dendritic cells at the tumor site, (ii) the reduction of MDSCs in patients and (iii) the reduction of tumoral mass.

Another ATRA-loaded liposome preparation was developed by Chen and colleagues and it comprised ATRA and a liposome carrier containing phospholipid, cholesterol and pegylated phospholipid (Patent n° CN107753427A; WO2018033118A1; EP3501500A1). The major advantage obtained was the increase in ATRA solubility due to the use of a combination of solubilizing molecule selected from PVP, HPMC, cyclodextrin and PEG. The preparation method adopted an active drug loading method which comprised a calcium acetate gradient method or a sodium acetate gradient method. The final preparation had not only a high drug loading but also a high stability in vivo, thereby improving drug plasma concentration and extending ATRA half-life. The preparation was advised also in the setting of a medicament for the oncology treatments and it was recommended for the injection preparation selected from subcutaneous, intravenous, intramuscular or pelvic injection types. This invention may be useful to improve the effect of ATRA on myeloid-inhibiting cells and on tumor-associated macrophages, as the preparation promoted MDSC differentiation into mature dendritic cells, MDSCs reduction and T cell proliferation at tumor site, finally inhibiting tumor proliferation and recurrence.

The worth remembering last invention provided an original ATRA nano-medicine preparation for oral hyperplasia and oral squamous cell carcinoma (OSCC) treatments. It included an ATRA medicine molecule, a PLGA-PEG nanocarrier encapsulating ATRA and a PD-L1 monoclonal antibody attached to nanocarrier surface (Patent n° CN110623942A). The choice of this antibody derived from the higher PD-L1 expression in patients with oral leukoplakia and oral squamous cell carcinoma compare to healthy people. Thanks to PD-1 monoclonal antibody position, outside the nano-carrier, the drug could reach the tumor site and reduce the drug dispersion. In vivo studies revealed the significant ability of this preparation to inhibit oral dysplasia and oral squamous carcinoma cell proliferation, thus finally promoting tumor cells apoptosis.

## 6. Conclusions

All in all, given the effective anti-cancer properties of ATRA, the development of novel delivery strategies to overcome the several limitations associated to therapy approaches with this drug represents an active field of research. In the past few years there was a burst of pre-clinical studies where several ATRA formulations were designed for different administrations aimed at ameliorating the current treatment of many tumors. Moreover, the trend observed for the pre-clinical studies is consistent with the increasing number of approved patents. Regrettably, none of these new formulations have been stepped into the clinics yet. Therefore, they have a long way to go before they are translated from bench-to-bed and they need a lot of effort but it worth it.

## Figures and Tables

**Table 2:** Patents related to ATRA delivery systems in cancer.

Novelty	Number of the Patent	Priority Date	Publication Date	National	International
Nano-fibular nanoparticles polymer-ATRA conjugate for sustained dermal delivery	WO2016210087A1; US2018185513A1	23rd June 2015	29th December 2016 (WO2016210087A1); 5th July 2018 (US2018185513A1)	USA	PCT
ATRA-loaded liposomal aerosols for delivery to the lungs	US6334999B1	27th August 1999	1st January 2002	USA	-
ATRA/TGFβ-loaded (PLGA) polymeric nanoparticles for the treatment of Type 1 Diabetes Mellitus	WO2015109245A1; US2016338984A1; US10105334B2	17th January 2014	23rd July 2015 (WO2015109245A1); 24th November 2016 (US2016338984A1); 23rd October 2018 (US10105334B2)	USA	PCT
ATRA quasicrystal-loaded liposomes for the treatment of solid tumors	CN109364027A	12nd December 2018	22nd February 2019	China	-
ATRA-loaded liposomes for the treatment of solid tumors	WO2018033118A1; CN107753427A; EP3501500A1	18th August 2016	22nd February 2018 (WO2018033118A1); 6th March 2018 (CN107753427A); 26th June 2019 (EP3501500A1)	China	PCT; European patent
ATRA/aPD-L1-loaded (PLGA-PEG) polymeric nanoparticles for the treatment of oral dysplasia and oral squamous carcinoma	CN110623942A	30th September 2019	31st December 2019	China	-
